# Improved Side Effect Profile of Alternate-Day Dosing of Lenalidomide

**DOI:** 10.7759/cureus.55317

**Published:** 2024-03-01

**Authors:** Ridwan A Lawal, Oluwole Banjoko, Chukwunonso Ndulue, Sodiq T Adebeshin, Arsalan Sharif, Osasumwen E Ighodaro, Rahman Olusoji, Bilikisu Odusanya, Nadia s El-Hamdi

**Affiliations:** 1 Internal Medicine, College of Medicine, University of Lagos, Lagos, NGA; 2 Internal Medicine, Lagos State University Teaching Hospital, Lagos, NGA; 3 Internal Medicine, University of Lagos, Lagos, NGA; 4 Medicine, Tbilisi State Medical University, Tbilisi, GEO; 5 Internal Medicine, Afe Babalola University, Ado Ekiti, NGA; 6 Internal Medicine, Columbia University at Harlem Hospital Center, New York, USA; 7 Pediatrics, Olabisi Onabanjo University, Sagamu, NGA; 8 Internal Medicine, Hospital Corporation of America (HCA) Houston Healthcare Kingwood, University of Houston College of Medicine (UHCOM), Texas, USA

**Keywords:** resource limited setting, alternate day dosing, lenalidomide, del(5q), myelodysplastic syndrome (mds)

## Abstract

Myelodysplastic syndrome (MDS) is a heterogeneous hematological condition associated with cytopenia, inadequate blood cell synthesis, and the risk of developing acute myeloid leukemia (AML). Patients are divided into risk groups according to the International Prognostic Scoring System (IPSS) to help direct therapy. Allogeneic stem cell transplantation, despite its limitations, is curative. Medical management, such as the use of lenalidomide, has potential benefits but can cause adverse effects that require dose regimen modification. Lenalidomide is approved for low-risk MDS with 5q deletion (5q- MDS). In this case study, a 79-year-old woman with 5q- MDS was switched from a daily regimen to an alternate-day lenalidomide dose schedule to achieve complete remission with fewer adverse effects. The management of hematological toxicity and the mechanisms of action of lenalidomide are discussed. We recommend individualized treatment strategies and additional research to improve MDS management.

## Introduction

Myelodysplastic syndrome (MDS) is a varied hematological disorder characterized by ineffective blood cell production, cytopenia, and abnormal cell morphology and carries a higher risk of developing acute myeloid leukemia (AML) [1]. Although some epidemiological data suggest the incidence of MDS may be increased in affluent regions, this remains a topic of debate. Most MDS cases result from randomly acquired genetic mutations [2].

Management of MDS starts with the use of a validated tool, such as the International Prognostic Scoring System (IPSS) or its revised version (IPSS-R), for risk assessment, which helps direct therapy. In lower-risk MDS (LR-MDS) cases, treatment focuses primarily on improving cytopenia, principally anemia. Conversely, in higher-risk MDS (HR-MDS) cases, treatment aims to impede disease progression and extend survival [3,4].

Myelodysplastic syndrome often requires a multifaceted and tailored range of therapeutic approaches. Among these, allogeneic hematopoietic stem cell transplantation is the sole potentially curative option, but it is only available to a limited number of medically fit patients. Treatment strategies for most individuals with MDS are less intensive and adjusted based on single- or multi-lineage cytopenia, risk (as determined by the IPSS or IPSS-R), as well as candidacy for targeted agents, which is determined by factors such as age, state of general health, and the presence of specific chromosomal aberrations or gene mutations. These strategies include hematopoietic growth factors, hypomethylating agents, and immunosuppressive therapy or immunomodulators (e.g., lenalidomide). These agents are not curative but improve quality of life by increasing blood cell counts, reducing the need for transfusions, and slowing disease progression [5].

Thalidomide, commonly used in hematologic malignancies over the past two decades, has shown some efficacy in MDS, but adverse effects, including sedation, constipation, neuropathy, and thromboembolism, result in patient discontinuation and limit its practical use. Lowering the daily dose is associated with fewer side effects but also with lower response rates [6]. Furthermore, the side effect profile of treatment may be more impactful in MDS than in other malignancies or multiple myeloma due to the somewhat older median age of MDS patients [7].

Lenalidomide, an oral thalidomide analog that down-regulates interleukin-6 and nuclear factor k-B and activates caspase 8, is up to 50,000 times as potent at inhibiting tumor necrosis factor alpha [7]. Additionally, lenalidomide dramatically upregulates the secreted protein acidic and rich in cysteine (SPARC) gene at 5q31-q32, which controls essential biological processes such as cell adhesion, proliferation, and differentiation via the regulation of interactions between cells and the extracellular matrix that surrounds them and preferentially suppresses the development of MDS deletion 5q (del(5q)) progenitors [8-12]. Lenalidomide is approved for treating patients with low- or intermediate-1-risk MDS with 5q deletion (5q- MDS) with transfusion-dependent anemia, with or without other cytogenetic abnormalities, which is particularly useful because these patients typically have poor responses to erythropoiesis-stimulating agents [8]. The recommended starting dose of lenalidomide in patients with 5q- MDS is 10 mg/day. However, significant myelosuppression may necessitate a dose reduction in many patients [6]. This report aims to raise awareness of the improved tolerance of lenalidomide for the treatment of 5q- MDS through an alternate-day dosing regimen instead of daily dosing to attain disease remission.

## Case presentation

A 79-year-old Nigerian woman with a past medical history of anemia and remote surgeries including caesarian section, appendectomy, and cataract surgery presented to the clinic for exhaustion and shortness of breath with minimal exertion for seven weeks. She experienced similar symptoms twice previously, each requiring hospitalization and three or more blood transfusions per hospitalization. A CT scan of the chest, endoscopy, and colonoscopy were normal then. She previously worked in an information department in Ghana and denied any radioactive exposure. She consumed a balanced diet and denied weight loss or appetite changes. She denied prescription or over-the-counter medications, smoking, and consuming alcohol.

**Figure 1:**
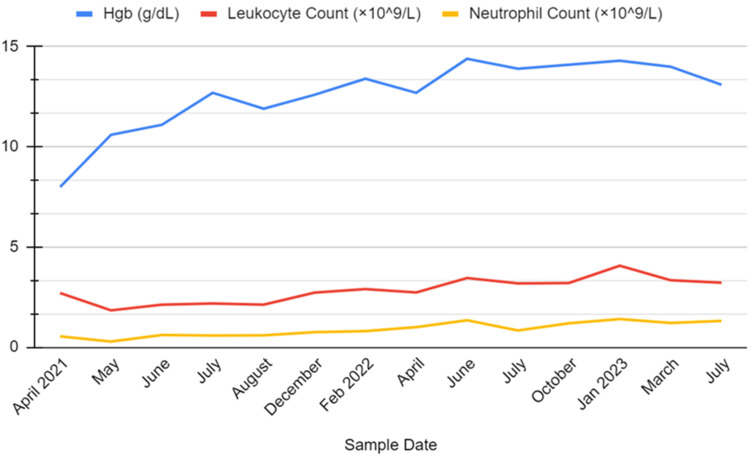
Hemoglobin, white blood cell count, and neutrophil count over 28 months

**Figure 2:**
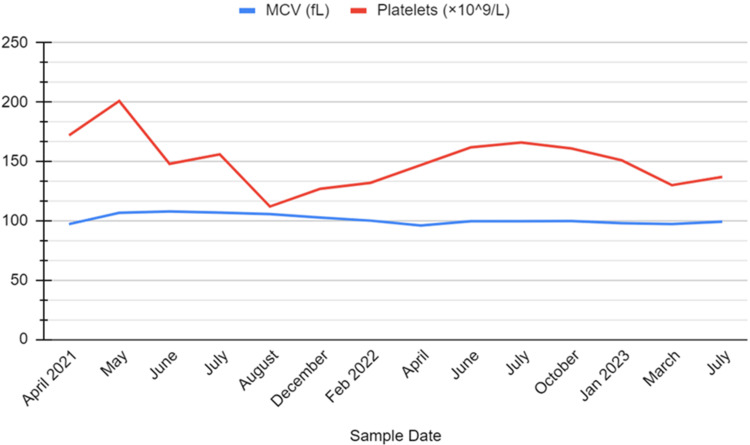
Platelets and MCV trend over 28 months MCV: Mean corpuscular volume

On physical examination, her vital signs were as follows: temperature, 37.2 Celsius; heart rate (HR), 120 beats/minute; respiratory rate (RR), 16 breaths/minute; O2 saturation, 94% on room air; and blood pressure, systolic 100 mmHg and diastolic 50 mmHg. Other pertinent exam findings included conjunctival pallor and delayed capillary refill. Complete cardiopulmonary, abdominal, and musculoskeletal exams were unremarkable. There were no rashes, bleeding, or signs of lymphadenopathy. Laboratory studies were remarkable for mild leukopenia, mild thrombocytopenia, elevated acute phase markers (B12, ferritin), macrocytic anemia, and acute kidney injury. These and additional laboratory investigations are detailed in Table 1. Liver function tests were normal. Abdominal ultrasound revealed no anomalies or acute findings. The fluctuations in her hemoglobin, white blood cell count, and neutrophil count over the 28-month period are depicted in Figure 1. Additionally, the variations in her platelet count and mean corpuscular volume (MCV) were similarly trended for 28 months and are illustrated in Figure 2.

**Table 1:** Laboratory workup at baseline and after two years of lenalidomide therapy HbsAg: Hepatitis B surface antigen; Hep C: Hepatitis C virus; eGFR: Estimated glomerular filtration rate; LFTs: Liver function tests

Parameters	Normal range	Result (on presentation)	Interpretation	Result (after two years on lenalidomide)
Hemoglobin (g/dL)	11.5-16.5	4.0	Severe anemia	14.0
Leukocytes (×10^9^/L)	4.0-11.0	3.0	Mild leukopenia	3.34
Neutrophil (%)	40-65	65	Normal	36.1
Lymphocyte (%)	20-40	23	Normal	48.7
Platelets (×10^9^/L)	150-450	149	Mild thrombocytopenia	130
Mean corpuscular volume (fL)	76-99	126	Macrocytic anemia	98
Peripheral blood film		Macrocytosis		
Fecal for occult blood		Negative		
HbsAg, Hep C antibody, HIV screening		Negative		
Serum vitamin B12 (pmol/L)	>138	1171	Elevated	
Serum ferritin (ƞg/ml)	22.0-112.0	541.2	Elevated	
Serum folate (ƞmol/L)	7.0-39.7	30.9	Normal	
Sodium (mmol/L)	135-150	151	Mildly elevated	
Potassium (mmol/L)	3.5-5.1	3.3	Mild hypokalemia	
Urea (mmol/L)	2.9-8.2	11.2	Elevated	
Creatinine (µmol/L)	80-115	116	Mildly elevated	
eGFR	>75	51.3 (70 at two-week follow-up)	Acute kidney injury	
Fluorescence in situ hybridization		Deletion 5q31 Deletion 5q32-33	Myelodysplastic syndrome with del5q (5q- MDS)	

**Figure 3:**
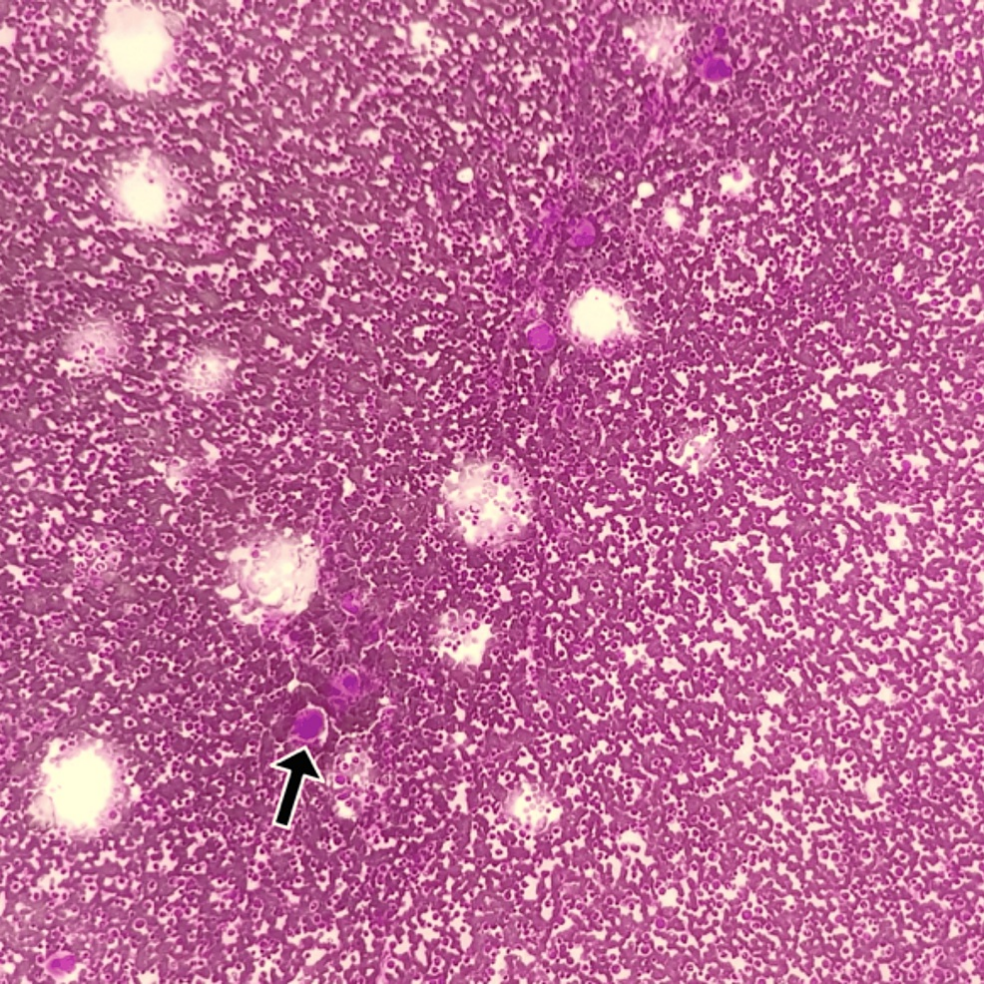
Histopathological image of the hypercellular marrow with increased megakaryopoiesis (black arrow), H&E stain, original magnification 10x H&E: Haematoxylin and eosin

**Figure 4:**
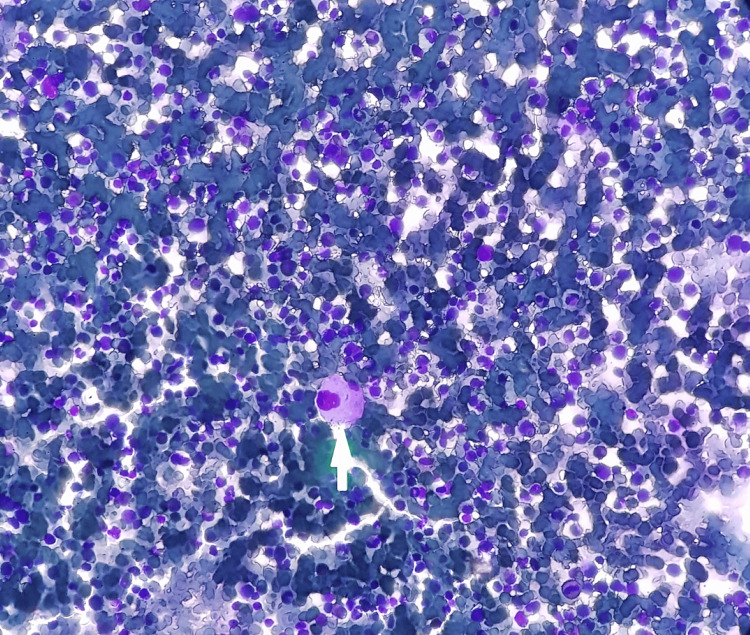
Hypercellular bone marrow aspirate with increased megakaryopoiesis, H&E stain, original magnification 40x The white arrow points to the megakaryocyte with a monolobated nucleus. H&E: Haemotoxylin and eosin

**Figure 5:**
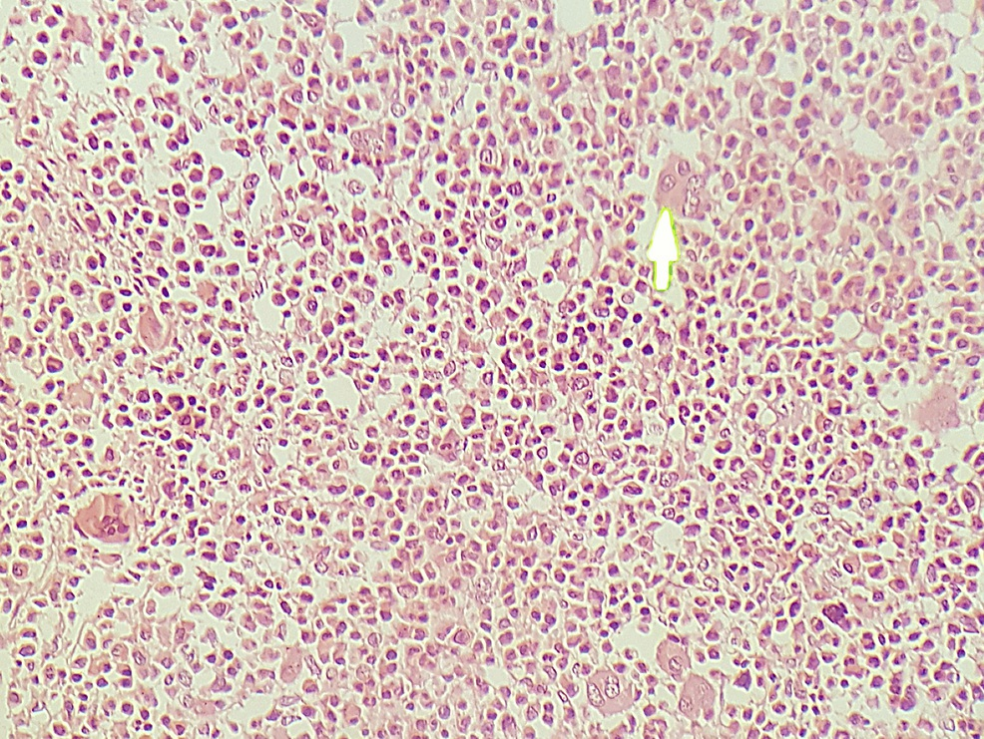
Increased megakaryopoiesis displayed on bone trephine H&E section, original magnification 40x The white arrow points to a megakaryocyte. H&E: Haemotoxylin and eosin

Blood products were transfused to optimize the packed cell volume, and our patient was transfusion-dependent until she began treatment. Bone marrow histology revealed hypercellular marrow and increased megakaryopoiesis (Figures 3, 4). Bone trephine yielded a specimen portraying similar results (Figure 5). Although focally observed, erythropoiesis was generally underrepresented, with serial maturation. Marrow fibrosis was increased, but no granulomatous inflammation was observed. Specimens sent for fluorescence in situ hybridization with specific DNA probes revealed a deletion of 5q31 in 67 of 165 examined cells (41%) and a loss of 5q32-33 in 54 of 113 (48%) interphase nuclei. Therefore, the diagnosis of 5q- MDS was established.

The patient was initiated on 10 mg daily lenalidomide with subsequent improvement in cell counts; however, she became neutropenic after the first cycle of lenalidomide, which resulted in medication discontinuation for two weeks. Subsequently, the neutrophil count increased as expected, and lenalidomide was restarted with a dosing frequency decreased to 10 mg on alternate days. This alternate-day regimen resulted in complete remission with no further leukopenia or neutropenia and a modest improvement in platelet count. Follow-up continued with a monthly complete blood count, and packed cell volume remained within the reference range between 39% and 42% in the following 28 months. Subsequent follow-up revealed the development of new-onset hypertension, for which she was prescribed 5 mg of amlodipine.

## Discussion

This is a unique case of a 79-year-old female diagnosed with MDS, characterized explicitly by deletion of the 5q chromosome (5q- MDS). The patient achieved complete remission with fewer adverse effects on an alternative-day lenalidomide dosing regimen. Although lenalidomide can safely treat individuals with lower-risk MDS, patients should be continuously monitored for neutropenia, thrombocytopenia, and leukopenia, and dosage adjustments are often necessary when administering lenalidomide [8,13].

Lenalidomide is an immunomodulator that affects cellular activities and reduces angiogenesis and inflammation, likely via CDC25C phosphatase inhibition, which controls cell cycle progression [11,14,15]. Despite the drug primarily targeting structures in the bone marrow microenvironment, it also directly affects clonal hematopoietic cells by inhibiting proteins critical for cell survival or stimulating tumor suppressor genes in the 5q region [15,16]. Additionally, lenalidomide enhances T-cell immune synergism by regulating the ratio of the T-helper 1 type to the T-helper 2 subset and regulating cytokine production [14,17]. However, several mechanisms of lenalidomide action on patients' hematopoiesis still need to be defined [11].

Our patient was initially prescribed 10 mg of lenalidomide daily, but treatment was discontinued due to neutropenia, a common adverse effect that limits the use of lenalidomide [18]. In a previous study, alternate-day lenalidomide showed promising results in 5q- MDS, with six patients achieving transfusion independence and some achieving cytogenetic responses. Mild toxicity was observed in a few cases, with manageable hematological adverse effects [18]. We intervened to mitigate these adverse effects while retaining therapeutic efficacy and modified the dosage to 10 mg on alternate days. This approach led to complete remission and aligned with existing literature recommending alternate-day dosing [19]. 

Our report further supports that lenalidomide is particularly effective in treating 5q- MDS. This may be attributable to its reduction of CDC25C levels and a positive impact on SPARC, but warrants further investigation. We present this case to raise physician awareness of alternative dosing regimens as efficacious treatment options using lenalidomide therapy via dose reduction or frequency reduction [20] to reduce hematologic toxicity in patients undergoing therapy for 5q- MDS. Further validation with a broader range of patients with MDS is needed. Additional studies may explore the impact of lenalidomide on other 5q- MDS biomarkers, such as SPARC, to identify potential treatment targets and the underlying cause. Furthermore, large, randomized controlled studies comparing daily, lower-dose, and alternate-day lenalidomide dosing could provide substantial evidence to optimize treatment.

## Conclusions

Overall, this report supports the growing evidence that complex hematologic disorders such as MDS require personalized treatment plans. Specifically, we found that dosing lenalidomide every other day can effectively treat 5q- MDS while reducing adverse effects. A tailored approach with regular monitoring and prompt dosage adjustments is recommended to optimize outcomes in MDS.
